# The Thermal Activity of Normal and Malignant Tissues

**DOI:** 10.1155/1998/45925

**Published:** 1998-12

**Authors:** Peter Mack, Li-Yao Cheng

**Affiliations:** 1 Department of Surgery Singapore General Hospital Outram Road Singapore 169608 Singapore; 2 Department of Biochemistry National University of Singapore 10 Kent Ridge Crescent Singapore 119260 Singapore

## Abstract

The usefulness of metabolic heat measurements in quantifying the response of a solid tumour to anticancer treatment was evaluated. The heat production characteristic of malignant tissues, as measured from human stomach, breast and liver cancer samples, was observed to be inconsistent, and its value could be higher or lower than that of its normal tissue of origin. The various thermal activity responses of an experimental rat hepatoma to hepatic artery ligation, cryotherapy, intra-arterial (i.a) Adriamycin (2.4 mg/ kg), i.a. Norcantharidin (0.5 mg/kg) were next studied. The *tumour/liver* (T/L) ratio of untreated tumour-bearing rats was 0.83 but this fell to a minimum at 24 h in both the hepatic artery ligation and the cryosurgery groups. In these two groups marked fluctuations in the heat production of normal liver occurred with poor recovery of the T/ L ratio even at 2--3 weeks. In the Adriamycin group, the T/L ratio dropped to a minimum at 5 days, and in the Norcantharidin group, at 3 days. Minimal disturbances in the thermal activity of liver tissue occured in these two chemotherapy groups and the T/L ratio recovered by 3 weeks. Norcantharidin appeared as efficacious as Adriamycin in the treatment of hepatoma when evaluated in terms of thermal activity.


*HPB Surgery*, 1998, Vol. 11, pp. 75-86
Reprints available directly from the publisher
Photocopying permitted by license only

© 1998 OPA (Overseas Publishers Association) N.V.
Published by license under
the Harwood Academic Publishers imprint,
part of The Gordon and Breach Publishing Group.
Printed in Malaysia.

### The Thermal Activity of Normal and Malignant Tissues

### Microcalorimetric Response of Liver Cancer to Hepatic Artery Ligation, Cryosurgery, Adriamycin and Norcantharidin

PETER MACK[a,*] and LI-YAO CHENG[b]

[a] *Department of Surgery, Singapore General Hospital, Outram Road, Singapore 169608, Republic of Singapore;*
[b] *Department of Biochemistry, National University of Singapore, 10 Kent Ridge Crescent, Singapore 119260, Republic of Singapore*

*(Received 12 December 1996; In final form 20 May 1997)*

**The usefulness of metabolic heat measurements in quantifying the response of a solid tumour to anticancer treatment was evaluated. The heat production characteristic of malignant tissues, as measured from human stomach, breast and liver cancer samples, was observed to be inconsistent, and its value could be higher or lower than that of its normal tissue of origin. The various thermal activity responses of an experimental rat hepatoma to hepatic artery ligation, cryotherapy, intra-arterial (i.a) Adriamycin (2.4 mg/kg), i.a. Norcantharidin (0.5 mg/kg) were next studied. The *tumour/liver* (T/L) ratio of untreated tumour-bearing rats was 0.83 but this fell to a minimum at 24 h in both the hepatic artery ligation and the cryosurgery groups. In these two groups marked fluctuations in the heat production of normal liver occurred with poor recovery of the T/L ratio even at 2–3 weeks. In the Adriamycin group, the T/L ratio dropped to a minimum at 5 days, and in the Norcantharidin group, at 3 days. Minimal disturbances in the thermal activity of liver tissue occured in these two chemotherapy groups and the T/L ratio recovered by 3 weeks. Norcantharidin appeared as efficacious as Adriamycin in the treatment of hepatoma when evaluated in terms of thermal activity.**

*Keywords:* Metabolic heat, Mylabris, Cantharidin, cryosurgery, ischaemia, microcalorimeter, hepatoma, Adriamycin

*Corresponding author.

### INTRODUCTION

Although constant efforts are made to develop better tests for cancer chemosensitivity [1–3], there has been little progress in methods of quantifying the efficacy of chemotherapy on solid tumours. Traditional methods involve the measurement of either the changes in the tumour growth patterns or the prolongation of host survival induced by the treatment [4]. There are, in addition, a wide variety of techniques which have been developed to allow the quantification of the viability of malignant cells in suspension prepared from animal tumours, such as the end-point dilution assays [5] and assays based on colony formation in vivo or in vitro [6]. However, all these require the tedious processes of removal of tumour cells from the original animal, dispersal into single-cell suspensions, enumeration of the cells, and finally, a test of the growth potential of the cells, either in new hosts or in cell culture. In addition, the potential drawback of all these suspension procedures is the danger of selecting a subpopulation that might not be typical of the entire tumour cell population.

A precise method for quantifying treatment efficacy of liver cancer by measuring the glucose oxidation of tumour tissue using a radio-tracer technique was previously developed by the author [7] and used for evaluating the efficacy of Norcantharidin in the treatment of hepatoma [8]. While that method was adequate in quantifying short term tumour response, it could become rather time-consuming and expensive if repeated measurements were to be used for monitoring tumour response on a long term basis. With these considerations in mind, it was decided to explore the use of thermal activity measurements as an alternative method for monitoring the long term response of an experimental hepatoma to various forms of anti-cancer treatment, including the drug Norcantharidin.

The present investigation is a sequel to the authors' previous study on the short term efficacy of Norcantharidin in hepatoma [8]. The objectives of the present study are four-fold: (1) to study the feasibility of using microcalorimetric measurements of tissue slices of different organs, both normal and malignant, as overall indices of their metabolic activities, (2) to study the inter-strain and inter-gender variations in thermal activity of rat liver tissue, (3) to determine the tumour/liver ratio of the thermal activity of an experimental hepatoma model in the rat, and (4) to compare the long term efficacy of intra-arterial Norcantharidin with three other treatment modalities, namely intra-arterial Adriamycin, hepatic artery ligation and cryosurgery in this experimental model using thermal activity as an index of tumour response.

### MATERIALS AND METHODS

### Animals

One hundred and sixty-seven rats weighing between 250 – 300 g (Laboratory Animal Centre, National University of Singapore) were used in this study. Of these, 17 were Wistar rats, another 10 were Sprague-Dawley rats, and the remaining 140 were of the inbred Buffalo strain. They were kept in cages in an air-conditioned environment at 25°C under a twelve-hour cycle of alternating darkness and illumination, fed a laboratory pellet diet (Wayne Food, UK), and given tap water ad libitum.

### Animal Anaesthesia

Intramuscular Ketamine at a dose of 7.5 mg/kg was as anaesthesia for all operative procedures on the rat.

### Cancer Cell Line

The cell line, Morris Hepatoma 7777, originally induced in the Buffalo rat [9] was purchased from American Type Cell Culture, Maryland, USA, grown in Ham's F12 nutrient medium (Sigma Chemical Co, St Louis, MO, USA), and used to produce the primary liver cancer model.

### Anti-cancer Drugs

Adriamycin in 10 mg vials was purchased from Farmitalia Carlo Erba Ltd, Herts, Italy. Norcantharidin were purchased in glass ampoules of 10 mg/2 ml from Beijing Fourth Pharmaceutical Works, China.

### Microcalorimeter

A commericial 2277 Thermal Activity Monitor System (TAM) with four operating channels (Thermometric AB, Järfälla, Sweden), was used to accurately measure the rate of heat production of tissue specimens. Each channel consisted of a twin thermopile heat conduction calorimeter into which insertion vessels could be introduced. The steel ampoule was loaded with 3 ml of the Hams F12 culture medium. The TAM

was coupled to a microcomputer and microcalorimetric data was collected with *Digitam 3*, a software package specially designed for data management and analysis with the TAM. As the TAM had four channels, it was possible to conduct concurrently, a maximum of 4 concurrent microcalorimetric measurements of a tissue sample at any one time.

### Sample Preparation

All tissue samples were immersed in F12 medium immediately upon harvesting. The sample weights were kept small and in the region of approximately 5 mg in order to avoid the effect of metabolic crowding [10]. A scalpel blade was used to slice the tissue sample into smaller pieces of approximately 0.3 mm thickness [11]. These tissue slices were next introduced into the steel ampoule and the caps tightened. The ampoule was then lowered into the calorimeter, allowed to equilibrate with the thermostated water bath and to generate a power curve on the monitor.

### Reproducibility of Measurements

To gauge the reproducibility of thermal measurements on tissue slices with the TAM, seven Wistar rats were used. From each animal, a tissue sample of either: (1) the small gut mucosa, (2) the heart, (3) the brain, (4) the stomach, (5) the spleen, (6) the gastrocnemius muscle or (7) the liver was harvested. Concurrent quadruplicate measurements were made on each of the seven tissue samples. From the power curves generated, the peak power in mJ/g wet wt was obtained and the coefficient of variation was calculated for each sample to evaluate the reproducibility of intra-sample measurements (Tab. I).

### Thermal Activity of Human Cancer Tissues

Fresh human cancer tissues were obtained from five patients in this study. From the first patient, punch biopsy specimens of a gastric adenocarcinoma and the adjacent normal stomach mucosa were obtained at endoscopy using a flexible fibreoptic gastroscope (Olympus, Japan). From the second and third patients who were both suffering from infiltrative ductal breast carcinoma, samples of malignant and adjacent breast tissues were harvested at mastectomy. Similarly, liver and liver cancer specimens were harvested at liver resection from the fourth and fifth patients who had hepatocellular carcinoma. Note was taken of the fact that the hepatocellular carcinoma of the fifth patient was angiographically embolized with metal coils 3 weeks prior to liver resection. All specimens were, upon harvesting, immediately immersed in Ham's F12 medium and transported to the laboratory within 5 minutes for thermal activity studies. From the measurements, the peak thermal power ($P_{max}$) expressed as mJ/g wet weight, was obtained for both normal and malignant tissues. The degree of activity of the various malignant tissues was then expressed as a *tumour/normal ratio*, a ratio of the thermal activity of tumour tissue to that of its corresponding tissue of origin (Tab. II).

### Thermal Activity of Normal Organ Tissues

In 30 male Buffalo rats, tissue samples of approximately 5 mg in size were harvested in duplicate from the gastrocnemius muscle ($n=4$), spleen ($n=3$), small gut ($n=6$), heart ($n=3$), stomach ($n=6$), brain ($n=3$) and liver ($n=5$) and their thermal activities were measured. In this and all subsequent studies using animal tissues, the results were expressed as the peak power in mJ/g dry weight instead of per g wet weight (Fig. 1). The dry weight could be calculated once the *wet wt./dry wt. ratio* was obtained. Since adequate animal tissues were available, it was possible to obtain the *wet wt./dry wt. ratio* of the various tissues by drying known weights of tissue samples in a hot air oven at 45°C for 3 weeks and re-weighing them after drying.

TABLE I Reproducibility of thermal activity measurements

| Organ of interest | Wt. of animal (g) | Wet wt. of tissue samples (mg) | Peak value of power curve (μJ) | Thermal activity (mJ/g wet wt) | Reproducibility of measurements (C.V.%) |
|---|---|---|---|---|---|
| Small gut | 300 | 4.9 | 18.73 | 3.82 | 8.3 |
| | | 4.6 | 16.16 | 3.51 | |
| | | 6.2 | 19.33 | 3.12 | |
| | | 6.1 | 20.87 | 3.42 | |
| Heart | 289 | 6.4 | 9.06 | 1.42 | 8.9 |
| | | 5.7 | 8.16 | 1.43 | |
| | | 5.9 | 8.39 | 1.42 | |
| | | 3.9 | 4.59 | 1.18 | |
| Brain | 275 | 5.8 | 23.46 | 4.04 | 5.6 |
| | | 4.9 | 21.50 | 4.39 | |
| | | 6.5 | 27.42 | 4.21 | |
| | | 6.3 | 24.27 | 3.85 | |
| Stomach | 281 | 3.8 | 11.21 | 2.95 | 10.4 |
| | | 4.5 | 14.10 | 3.13 | |
| | | 7.7 | 18.84 | 2.45 | |
| | | 4.4 | 13.25 | 3.01 | |
| Spleen | 286 | 5.5 | 13.72 | 2.49 | 5.4 |
| | | 6.1 | 17.27 | 2.83 | |
| | | 7.7 | 20.53 | 2.67 | |
| | | 7.8 | 20.18 | 2.59 | |
| Muscle | 288 | 4.6 | 1.84 | 0.40 | 14.6 |
| | | 5.4 | 2.16 | 0.40 | |
| | | 6.5 | 3.46 | 0.53 | |
| | | 6.4 | 2.65 | 0.41 | |
| Liver | 250 | 4.6 | 21.32 | 4.63 | 4.2 |
| | | 4.1 | 19.96 | 4.87 | |
| | | 5.0 | 24.12 | 4.82 | |
| | | 6.0 | 26.63 | 4.44 | |

TABLE II Thermal activity of some Human cancers

| Patient no. | Organ of malignancy & histology | Tissue sample | Thermal activity mJ/g wet wt. | T/N ratio (Tumour/Normal) |
|---|---|---|---|---|
| 1 | Stomach | Tumour | 2.85 | 2.2 |
| | adenocarcinoma | Normal stomach | 1.31 | |
| 2 | Breast | Tumour | 0.11 | 2.8 |
| | infiltrative ductal carcinoma | Normal breast | 0.04 | |
| 3 | Breast | Tumour | 0.90 | 2.6 |
| | infiltrative ductal carcinoma | Normal breast | 0.34 | |
| 4 | Liver | Tumour | 0.95 | 0.29 |
| | hepatocellular carcinoma | Normal liver | 3.33 | |
| 5 | Liver | Tumour (*embolized*) | 0.25 | 0.10 |
| | hepatocellular carcinoma | Normal liver | 2.62 | |

In this particular experiment, measurements of the total amount of thermal power (mW/g dry wt.) generated in the first hour by the various tissue samples, as determined by the area under the first hour of the power curve were also made. This enabled the correlation of the values of the peak power and the energy dissipated during the first hour to be statistically studied.

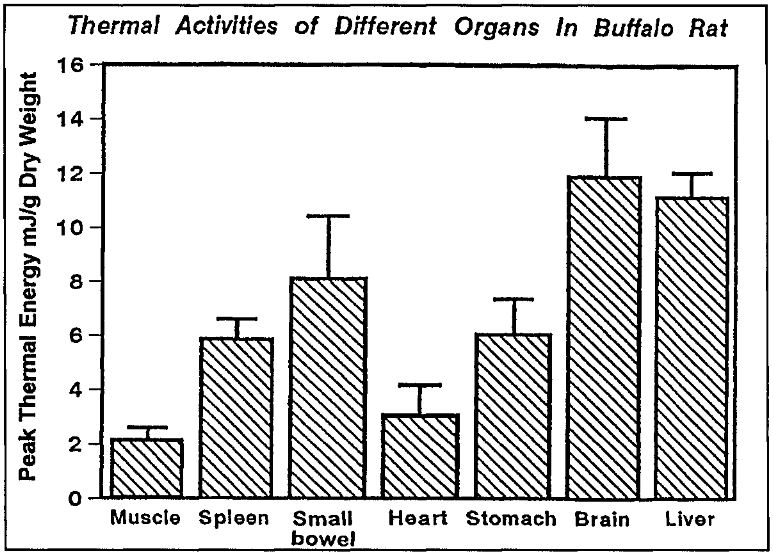

FIGURE 1 Thermal activity of various body organ tissues in the Buffalo rat expressed as the peak energy of the power curve in mJ per gram dry weight of the tissue. Vertical bars represent standard deviations.

### Thermal Activity of Normal Rat Liver Tissues

Thirty-two rats with equal numbers of male and female animals were used to study the inter-strain and inter-gender variations of the thermal activity of the liver. Of the 32 animals, 10 were Wistar, another 10 were Sprague-Dawley and the remaining 12 were Buffalo rats. They were anaesthetised, and liver biopsies of approximately 5 mg were harvested via a midline laparotomy. Measurements of their peak power were done in duplicates on the TAM and results were expressed as mJ/g dry weight.

### Primary Liver Cancer Model

The Morris Hepatoma 7777 cell line was used to produce a solid tumour model. The cells were cultured in Ham's F12 nutrient medium in an incubator at 37°C at 95% oxygen and 5% carbon dioxide for 4 days following which the cells were harvested with 0.25% trypsin (Cytosytems Co, Castle Hill Blue, Aust). Viability counts were made after staining with trypan blue (Sigma Chemical Co. USA). The tumour cells were then resuspended in Ham's F12 medium and $1.5 \times 10^6$ viable cells/0.1 ml were injected intraparenchymally into the edge of the left lateral lobe of the liver via a midline laparotomy. The abdomen was closed with a 3'O' Vicryl suture and the animal allowed to recover from anaesthesia. After two weeks, by which time the liver tumour would have grown to a size of $1-2$ $cm^3$, the inoculated animals were ready for experiments.

### Liver Cancer Experiments

A total of 90 male hepatoma-bearing Buffalo rats were used for this series of experiments. As a baseline, 6 untreated male tumour-bearing Buffalo rats were sacrificed and samples of their tumour and liver tissue of approximately 5 mg in weight were harvested in duplicate for thermal activity studies. The results obtained from this group of animals were designated as 0 h, which is the baseline control for the four subsequent experiments as follows:

### *(a) Hepatic Artery Ligation Group*

In a first group of 28 male tumour-bearing Buffalo rats, a midline laparotomy was performed and the falciform ligament and all peritoneal attachments to the liver and the hepatic branch of the hepato-oesophageal artery were divided. The proper hepatic artery, the continuation of the common hepatic artery after giving off the gastroduodenal artery, was identified and ligated by a 6 'O' silk suture. The laparotomy incisions were closed by a 3 'O' Vicryl suture and the animals were sacrificed at 1 h, 24 h, 3 days, 5 days, 7 days, 10 days, 14 days and 21 days after interruption of hepatic arterial blood flow. Upon sacrifice, tissue specimens of approximately 5 mg each were harvested immediately from the tumour and adjacent liver parenchyma and were subjected to microcalorimetric measurements in duplicate.

### *(b) Cryosurgery Group*

In a second group of 19 male tumour-bearing Buffalo rats, a midline laparotomy was performed under anaesthesia and the liver tumour was subjected to cryosurgical therapy. Cryofreezing was performed with the aid of Tissue-Tek (Miles, Elkhart, USA), which is an embedding medium for frozen tissue specimens containing 10.24% w/w polyvinyl alcohol, 4.26% w/w polyethylene glycol and 85.5% w/w non-reactive ingredients. The Tissue-Tek was immersed in liquid nitrogen and frozen into a bolus of approximately 1 cm in diameter. This frozen bolus was applied directly over the surface of the liver tumour for a few seconds and allowed to soften slightly to fit the upper convex surface of the tumour, thus freezing the tumour by contact. The liver tumour was maintained in the frozen state by repeated applications of liquid nitrogen, dropwise from a 3 ml Pasteur pipette onto the bolus Tissue-Tek. Care was taken to ensure that the area of freezing was confined as much as possible to the area of the tumour tissue. After 20 minutes of freezing, the tumour tissue was allowed to thaw. The laparotomy incision was closed by 3 'O' Vicryl and the animals were allowed to recover from anaesthesia in a warm environment under the Vicker Medical Radiant Warmer (Model 183, UK). Post-operatively, the rats were sacrificed at 1 h, 24 h, 3 days, 5 days, 7 days, 10 days, 14 days and 21 days. Both tumour and liver tissues were harvested for microcalorimetric measurements.

### *(c) Intra-arterial Adriamycin Group*

In the remaining two groups of the experiment, all tumour-bearing rats underwent anaesthesia and a midline laparotomy to expose the gastroduodenal branch of the common hepatic artery. A fine polyethylene cannula (Portex, 800/100/100/100, UK) filled with normal saline, was inserted under an operating microscope in a retrograde direction into the gastroduodenal artery and secured in position by a 6 'O' silk ligature.

In the third group of tumour-bearing rats ($n = 21$), a bolus dose of intra-arterial Adriamycin at 2.4 mg/kg in 0.3 ml saline was infused over 5 minutes and the cannula was flushed with a further infusion of 0.5 ml of normal saline.

### *(d) Intra-arterial Norcantharidin Group*

In the fourth group of animals ($n = 16$), a single bolus dose of 0.5 mg/kg of intra-arterial Norcantharidin was similarly given in each animal. All laparotomy wounds were closed with 3 'O' Vicryl and all animals were sacrificed at 1 h, 24 h, 3 days, 5 days, 7 days, 10 days, 14 days and 21 days after intra-arterial drug administration. Upon sacrifice, tissue specimens of approximately 5 mg each were harvested in duplicate immediately from the tumour and adjacent liver parenchyma for measurements in the microcalorimeter. The results of this and all the 3 other groups in this series of experiment were expressed as mJ/g dry wt.

### Statistics

All results were expressed as mean ± standard deviation. Analysis of variance (anova) and the Student's *t*-test were used for analysis of differences between groups.

### RESULTS

### Reproducibility of Measurements

The coefficient of variation obtained through quadruplicate measurements of a single sample was 8.3 % for small gut, 8.9 % for heart, 5.6 % for brain, 10.4 % for stomach, 5.4 % for spleen, 14.6 % for muscle and 4.2 % for liver.

### Human Cancer Tissues Studies

The pattern of thermal activity of the human cancers studied is shown in Table II. Of interest, the stomach and breast cancer specimens produced more heat than their corresponding tissues of origin, whereas the reverse was true for primary liver cancer. When expressed as a ratio of the heat production of tumour tissue over that of the normal (*T/N ratio*), the tumour activity was 2.2 in the patient with stomach cancer, 2.8 and 2.6 respectively, in the two breast cancer patients, 0.29 in one patient with liver cancer and 0.10 in the other patient whose liver cancer was embolized three weeks prior to liver resection.

### Normal Organ Tissue Studies

Thermal activity measurements showed that, the peak power ($P_{max}$) of the various tissues studied were, in descending order, 11.91 ± 2.12 mJ/g dry wt. for brain, 11.17 ± 0.85 mJ/g dry wt. for liver, 8.05 ± 2.30 mJ/g dry wt. for small gut, 6.08 ± 1.25 mJ/g dry wt. for stomach, 5.84 ± 0.74 mJ/g dry wt. for spleen, 3.03 ± 1.10 mJ/g dry wt. for heart muscle, and 2.09 ± 0.49 mJ/g dry wt. for skeletal muscle (Fig. 1). The values of the $P_{max}$ were correlated with their respective areas under the power curve for the first hour, which represented the total energy dissipated by the tissue specimen in the first hour (Fig. 2). A highly significant correlation was obtained ($r = 0.97$, $p < 0.001$).

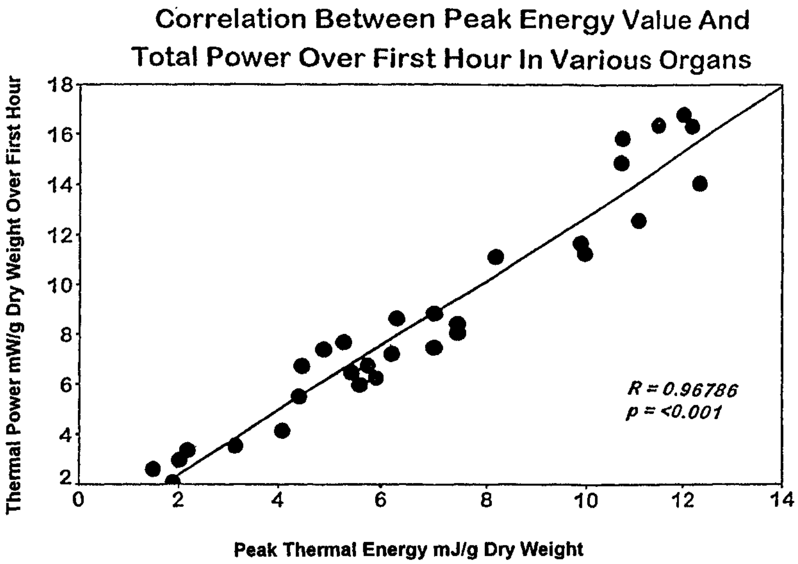

FIGURE 2 Correlation between the area under the power curve (total thermal power in mW/g dry wt.) for the first hour with the corresponding peak energy of the power curve $P_{max}$ in mJ/g dry wt. ($r$=0.97, $p$ < 0.001).

### Normal Liver Studies

Thermal activity measurements of the rat liver tissues showed no differences in heat production amongst the males in the three strains, Wistar, Sprague-Dawley and Buffalo (Tab. III). However, a significant difference did exist among the female rats between the three strains (Anova, $p < 0.005$). In all 3 rat strains, the liver tissue of the males showed a significantly higher thermal activity than that of the females, $p < 0.00005$ for Buffalo, $p < 0.00001$ for Wistar and $p < 0.05$ for Sprague-Dawley.

### Liver Tumour Experiments

The baseline *tumour/liver* (T/L) ratio of untreated tumour-bearing rats was 0.83 (Tab. IV). This T/L ratio dropped by one third to a level of 0.56 in the first hour after hepatic artery ligation. The ratio fell to a minimum level of 0.12 (14% of baseline) at 24 h. The thermal activity of the liver fluctuated considerably during the first week of treatment but became more stable subsequently. Recovery was slow and incomplete, only upto 0.52 at 3 weeks.

TABLE III Thermal activity of normal liver tissue of various rat strains

| Rat strain | Sex | No. of animals ($n$) | Thermal activity mJ/g dry wt. | $P$ value |
|---|---|---|---|---|
| Buffalo | Male | 6 | 15.58 ± 0.45 | < 0.00005 |
| | Female | 6 | 13.27 ± 0.72 | |
| Wistar | Male | 5 | 15.46 ± 0.43 | < 0.00001 |
| | Female | 5 | 12.34 ± 0.50 | |
| Sprague-Dawley | Male | 5 | 15.26 ± 1.02 | < 0.05 |
| | Female | 5 | 13.92 ± 0.58 | |

In the cryosurgery group, the T/L ratio dropped by half to 0.40 and then to a minimum of 0.14 (17% of baseline) at 24 h. There was marked fluctuation of the thermal activity of the liver tissue in the cryosurgery group after treatment (Fig. 3) and none of the treated animals could survive up to 21 days to complete the protocol. Of those that survived 14 days after cryosurgery, the ratio came up to only 0.66.

In the Adriamycin group, the T/L ratio also dropped by approximately half in the first hour to 0.43. Further drop was prolonged and the minimum of 0.22 (27% of baseline) was reached at 5 days. Recovery in the Adriamycin-treated group was however complete at 3 weeks.

In the Norcantharidin group the T/L ratio dropped by a quarter to 0.63 in the first hour after treatment. The ratio reached a minimum of 0.22 (27% of baseline) at 3 days. Recovery in the Norcantharidin group was also complete by 3 weeks. In both the Adriamycin and Norcantharidin groups, the thermal activities of the liver were noted to relatively stable throughout the entire experiment (Fig. 4).

### DISCUSSION

Microcalorimetry has the capacity of a general analytical tool for the quantitative assessment of

TABLE IV Changes in T/L ratios following various anti-hepatoma treatments in rat model

| Groups | Tumour/Liver (T/L) ratios at various time points after treatment 0 hr | 1 hr | 24 hr | 3 days | 5 days | 7 days | 10 days | 14 days | 21 days |
|---|---|---|---|---|---|---|---|---|---|
| Hepatic artery ligation | 0.83 ($n$ = 6) | 0.56 ($n$ = 4) | 0.12 ($n$ = 4) | 0.25 ($n$ = 3) | 0.26 ($n$ = 3) | 0.20 ($n$ = 4) | 0.27 ($n$ = 3) | 0.55 ($n$ = 3) | 0.52 ($n$ = 4) |
| Cryosurgery (20 min) | 0.83 ($n$ = 6) | 0.40 ($n$ = 4) | 0.15 ($n$ = 3) | 0.19 ($n$ = 2) | 0.08 ($n$ = 3) | 0.57 ($n$ = 3) | 0.55 ($n$ = 2) | 0.66 ($n$ = 2) | – |
| I/A Adriamycin (2.4 mg/kg) | 0.83 ($n$ = 6) | 0.43 ($n$ = 2) | 0.24 ($n$ = 2) | 0.23 ($n$ = 2) | 0.21 ($n$ = 4) | 0.29 ($n$ = 2) | 0.70 ($n$ = 3) | 0.75 ($n$ = 3) | 0.80 ($n$ = 3) |
| I/A Norcantharidin (0.5 mg/kg) | 0.83 ($n$ = 6) | 0.63 ($n$ = 2) | 0.28 ($n$ = 2) | 0.22 ($n$ = 2) | 0.26 ($n$ = 2) | 0.34 ($n$ = 2) | 0.71 ($n$ = 2) | 0.94 ($n$ = 2) | 0.81 ($n$ = 2) |

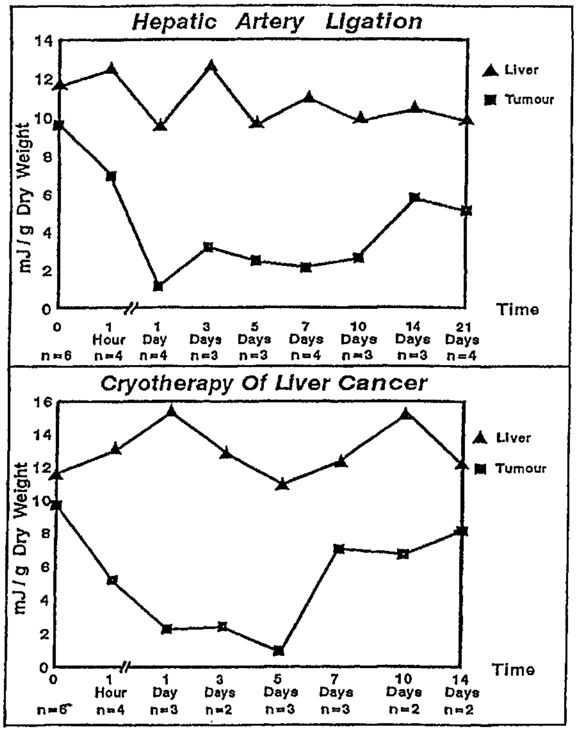

FIGURE 3 The tumour/liver ratio of heat production in hepatoma-bearing animals, subjected to hepatic artery ligation (in the top half of the graph) and to cryosurgery (in the bottom half of the graph. Note the degree of fluctuation in liver activity in both groups.

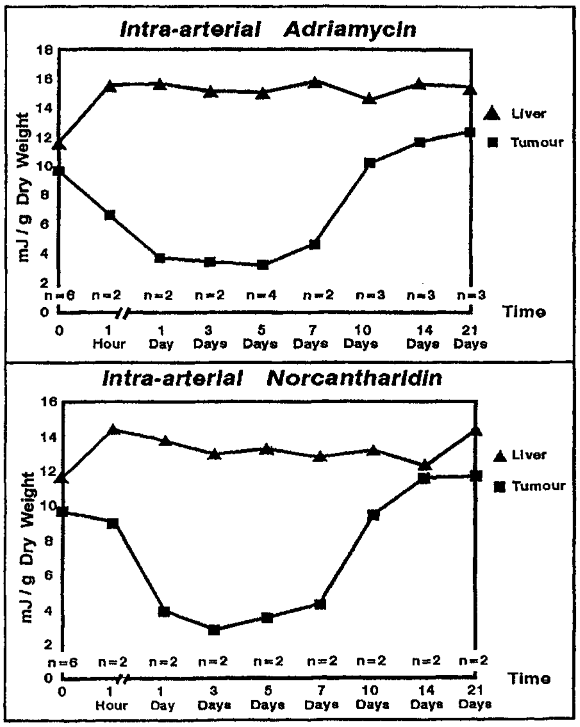

FIGURE 4 The tumour/liver ratio of heat production in hepatoma-bearing animals treated with i.a. Adriamycin at 2.4 mg/kg (in the top half of the graph) and with i.a. Norcantharidin at 0.5 mg/kg (in the bottom half of the graph). The fluctuations in liver tissue activity were minimal and closer to a horizontal straight line when compared to that of Figure 3.

the overall physical and chemical changes of many living cellular systems [12] including the study of the process of microbial metabolism, the evaluation of erythrocytes in hyperthyroid states, and even for the study of isolated hepatocytes [13]. Modern microcalorimetry has the advantages of good sensitivity and easy automation, making it an excellent method for observation of the overall metabolic activity of cell processes. Knowledge of the difference in heat production between normal and neoplastic cells led to the application of the microcalorimetric method in the monitoring of the response of leukaemia and lymphoma patients to treatment, [14–16] and subsequently in the prediction of sensitivity of neoplastic cells to chemotherapy [17, 18].

An extension of the use of microcalorimetry from pure cellular systems to living tissue slices enabled the technique to be used for monitoring small changes in tissue metabolism occurring in in-vivo systems. Asakawa and his colleagues [19, 20] successfully applied the tissue-slice technique to small liver biopsy specimens and was able to repeatedly quantify the heat production of liver tissues biopsied from living rats undergoing hepatic ischaemia and re-perfusion studies. In this study, the authors extended this application successfully for the monitoring of heat production changes in experimental liver cancer undergoing various forms of anti-cancer treatment. The results in this study indicated potential usefulness of metabolic heat measurements as a bioassay of tumour activity. As a result, the authors were able to adequately assess the long term efficacy of Norcantharidin, a relatively unknown anti-cancer drug, in comparison with conventional modalities of anti-hepatoma treatment.

An initial pilot study of the various major solid organ tissues of the body showed that the liver had by comparison, one of the highest level of thermal activity (Fig. 1) with good reproducibility of measurements (Tab. I). This rendered the microcalorimetric method appropriate for evaluating changes in the thermal activities of liver and liver tumours with anti-cancer treatment. The high correlation between the peak activity and area under the power curve over the first hour enabled the authors to conveniently adopt the peak value as a quick and reliable indicator for monitoring cancer tissue activity. Of note, the microcalorimetric method has been found sufficiently sensitive to detect subtle inter-gender as well as inter-strain differences in rat liver tissue activity.

Although it is common knowledge that the metabolism of most malignant tissues were higher than their normal tissues of origin because of exaggerated glycolysis [21], metabolic heat measurements in this study did not consistently show exaggerated heat production of malignant tissues over their normal counterparts as expected. Of the human cancer specimens examined, heat production from both the gastric and breast carcinoma tissues were found to be more than twice that of their normal counterparts, whereas hepatocellular carcinoma tissue was only 0.3 that of normal liver tissue. The tumour/liver ratio in the patient with an embolized liver tumour was noted to be three times as low as the patient without embolization. This last observation however, was consistent with the authors' surmise that metabolic heat measurement could be useful as a bioassay for tumour activity, before and after treatment.

In the authors' previous study, it was shown that the glucose oxidation rate of the rat Morris Hepatoma model (or T/L ratio) was 4.2 times that of normal liver tissue [8]. Yet, in the present study where thermal activity was used as an index of tumour activity, the T/L ratio was only 0.83. This apparent paradox can be explained by the fact that heat production measured in such situations did not represent merely the heat generated from glycolysis alone, but the sum activity of the total physico-chemical processes of the biological system that resulted in heat dissipated to the surrounding medium. Of interest, Monti [15] similarly noted that the heat production in peripheral blood lymphocytes of chronic lymphocytic leukaemia patients was also lower than that of lymphocytes of healthy subjects.

In the palliative treatment of non-resectable primary liver cancers, a variety of therapeutic modalities are currently available. Surgical ligation of the hepatic artery is a traditional method of proven value, but is a once-off treatment with only short-term efficacy because the liver tumour will regain its arterial supply through collaterals some weeks later [22]. Cryosurgical therapy of liver cancers is a relatively new modality, and has shown encouraging results. However, since it is conducted intra-operatively it is also an once-off treatment, but it is possible to combine its use with intra-arterial chemotherapy. In the present study, both these two physical methods of treatment were shown to be more potent as compared with the two forms of intra-arterial chemotherapy. Their maximum effects were seen in 24 hours compared to 3 days in Norcantharidin and 5 days in Adriamycin. In addition, their recovery were also delayed as compared to the two intra-arterial chemotherapy groups in which recovery was complete within 3 weeks. The constitutional disturbance caused by the cryotherapy as reflected by the significant fluctuations in the values of heat production of the post-treated liver, probably accounted for the mortality of the animals noted in this group.

Adriamycin is a well and long established chemotherapeutic agent for primary hepatocellular carcinoma, but the use of Norcantharidin in this disease is much less well known. Norcantharidin is the demethylated form of Cantharidin, which is the active ingredient of the blister beetle, Mylabris, a long used Chinese traditional medicine. Cantharidin had been shown in China to have an effect on primary hepatoma, but the use was limited by its severe toxicity for the mucuous membranes of the gastrointestinal and urinary tract [8]. The analogue Norcantharidin was subsequently developed and it is known to possess significant anti-hepatoma activity but at the same time relatively free from side-effects, including bone marrow suppression [23]. In an earlier study by the author [8], it was shown that, when given by the intra-arterial route, Norcantharidin suppressed oxidative glucose metabolism by nearly 50% within one hour of administration. The present study showed that the heat production dropped by only 75% within the first hour but it exerted its maximum effect within 3 days of administration and recovery was complete by 3 weeks.

In conclusion, thermal activity measurement by microcalorimetry is a precise and convenient method of monitoring the long term response of an experimental hepatoma to treatment. The efficacy of physical modes of therapy in the form of arterial ischaemia and cryotherapy appeared more intense and to have a faster onset, when compared to the chemotherapeutic forms of treatment using Adriamycin and Norcantharidin. Norcantharidin, when given intra-arterially in this study, appeared to be at least as efficacious as Adriamycin. The information obtained herein also provided some clues on the pharmacodynamics of intra-arterial Norcantharidin on a hepatoma model, and the knowledge thus acquired might help to improve scheduling of chemotherapy regimens in the future.

### Acknowledgments

The authors wish to thank: (1) Dr. Arne Schön of Thermometric AB, Järfälla, Sweden for his guidance on the use of the Thermal Activity Monitor, (2) Mr. Robert Ng, Ms. In-Ching Song and Ms Irene Kee of the Department of Experimental Surgery, Singapore General Hospital for their technical assistance, and (3) Professor Guang-Sheng Wang of Beijing Medical University, China for contributing his expert knowledge on Norcantharidin.

This project was supported by a research grant from the Singapore Totalisator Board.

### References

[1] Durkin, W. J., Ghanta, V. K., Balch, C. M., Davis, D. W. and Hiramoto, R. N. (1979). A methodological approach to the prediction of anticancer drug effect in humans. *Cancer Res.*, **39**, 402–407.

[2] Weisenthal, L. M., Marsden, J. A., Dill, P. L. and Macalusa, C. K. (1983). A novel dye exclusion method for testing in vitro chemosensitivity of human tumors. *Cancer Res.*, **43**, 749–757.

[3] Salmon, S. E. (1984). Development and applications of a human tumor colony assay for chemosensitivity testing. *Rec. Res. Cancer Res.*, **94**, 8–16.

[4] Kallman, R. F. and Rockwell, S. (1975). Effects of radiation on animal tumor models. In *Cancer–A comprehensive treatise*, edited by F. F. Becker, **3**, 317–392, New York, Plenum Press.

[5] Hewitt, H. B. (1953). Studies of the quantitative transplantation of mouse sarcoma. *Brit. J. Cancer*, **7**, 367–383.

[6] Shaeffer, J., Mahdi, A. M. and Constable, W. C. (1973). Lung colony assays of murine mammary tumor cells irradiated in vivo and in vitro. *Radiology*, **109**, 703–706.

[7] Mack, P., Ahrén, B., Jeppsson, B., Zuxing, K. and Bengmark, S. (1988). Influence of 2-deoxy-d-glucose and arterial ischaemia on glucose oxidative metabolism and growth of liver cancer. *Eur. J. Cancer Clin. Oncol.*, **24**, 1433–1437.

[8] Mack, P., Ha, X. F. and Cheng, L. Y. (1996). Suppression of $^{14}C$-labelled tumour glucose oxidative metabolism by Norcantharidin in rat Morris Hepatoma. *HPB Surgery*, **10**, 65–72.

[9] Morris, H. P., Dyer, H. M., Wager, B. P., Miyaji, H. and Rechcigi, M.JR. (1964). Some aspects of the development, biology and biochemistry of rat hepatomas of different growth rate. *Adv. Enzyme Regul.*, **2**, 321–333.

[10] Sand, T., Condie, R. and Rosenberg, A. (1977). Metabolic crowding effect in suspension of cultured lymphocytes. *Blood*, **50**, 337–346.

[11] Umbreit, W. W., Bujrris, R. H. and Stauffer, J. F. (1964). *Manometric Techniques–A manual describing methods applicable to the study of tissue metabolism*, 4th Edu. Burgess Publishing Company.

[12] Beezer, A. E. (1980). *Biological Microcalorimetry*, London, Academic Press.

[13] Jarrett, I. G., Clark, D. G., Filsell, O. H., Harvey, J. W. and Clark, M. G. (1979). The application of microcalorimetry to the assessment of metabolic efficiency in isolated rat hepatocytes. *Biochem. J.*, **180**, 631–638.

[14] Schön, A. and Wadsö, I. (1986). Thermochemical characterization of T-lymphoma cells under non-growing conditions. *Cytobios*, **48**, 195–205.
[15] Monti, M., Brandt, L., Ikomi-Kumm, J., Olsson, H. and Wadsö, I. (1981). Metabolic activity of Lymphoma cells and clinical course in Non-Hodgkin Lymphoma (NHL). *Scan. J. Haematol.*, **27**, 305–310.
[16] Ankerst, J., Faldt, R. and Monti, M. (1984). Decreased responsiveness to immune complexes of granulocytes from patients with acute leukemia in remission demonstrated by microcalorimetry. *Leukaemia Res.*, **8**, 997–1002.
[17] Kiumura, T., Schön, A. and Wadsö, I. (1990). Prediction of the cytotoxic effects of some antineoplastic drugs on cultured T-lymphoma cells by microcalorimetry. *Cytobios*, **63**, 7–13.
[18] Schön, A. and Wadsö, I. (1988). The potential use of microcalorimetry in predictive tests of the action of antineoplastic drugs on mammalian cells. *Cytobios*, **55**, 33–39.
[19] Asakawa, H., Nässberger, L. and Monti, M. (1990). Microcalorimetric studies on metabolism of hepatic tissue. I. A methodological study of normal tissue. *Res. Exp. Med.*, **190**, 25–32.
[20] Asakawa, H. and Nässberger, L. (1990). Microcalorimetric studies on metabolism of hepatic tissue. II. Measurements of Ischemic Tissue. *Res. Exp. Med.*, **190**, 33–41.
[21] Warburg, O. (1930). *The metabolism of tumours*, London: Constable and Co.
[22] Mack, P., Jeppsson, B., Rajszys, P., Kobayashi, H., Asakawa, H. and Bengmark, S. (1989). Retarding liver cancer growth in the rat by transient repeated hepatic dearterialisation. *J. Surg. Res.*, **46**, 123–128.
[23] Wang, G. S. (1989). Medical uses of Mylabris in ancient China and recent studies. *J. Ethnopharmacol.*, **26**, 147–162.

### COMMENTARY MANUSCRIPT THE THERMAL ACTIVITY OF NORMAL AND MALIGNANT TISSUES– MICROCALORIMETRIC RESPONSE OF LIVER CANCER TO HEPATIC ARTERY LIGATION, CRYOSURGERY, ADRIAMYCIN AND NORCANTHARIDIN

This is an experimental study on the usefulness of metabolic heat measurements in quantifying tumour response to anti-cancer treatment. The increased heat production has been measured mostly in malignancies of hematopoetic and lymphatic systems and shows a good correlation to long term prognosis and response to therapy. However, its use in similar evaluation in solid tumours have not been studied much since there are some methodologic errors. Its use in the study of malignant tumours of the GI tract is hampered by the presence of bacteria which may have a significant influence on the heat production in the small tissue sections that are used. Its use in organs outside the GI mucosa, e.g., liver as well as breast tissue is interesting. In this paper the authors have shown some correlation with reduction of heat production in liver tumours after treatment with hepatic artery ligation or cryosurgery. Similar findings were observed after treatment with adriamycin or norcantharidin. There is, however, a fairly wide variation and this may indicate that the technique needs further refinement. The authors have also included some human data showing that this is a technique that is applicable in the clinic. Here the authors show that tumours of the stomach and breast have a higher thermal activity than normal tissue but in the liver with hepatocellular carcinoma the opposite is found. This is in contrast to the experimental study but one of the tumours was treated with embolization and thus data for only one clinical observation remain. Therefore the interpretation must be performed with caution. May be the hepatocellular carcinoma is unique among the tumours studied it mostly is highly differentiated and the difference to normal tissue may not be so pronounced.

Prof. B. W. Jeppsson
Department of Surgery
Malm University Hospital
Malm
S-205 02
Sweden

